# Long‐term demyelination and aging‐associated changes in mice corpus callosum; evidence for the role of accelerated aging in remyelination failure in a mouse model of multiple sclerosis

**DOI:** 10.1111/acel.14211

**Published:** 2024-05-28

**Authors:** Elham Parandavar, Mahshid Shafizadeh, Shahin Ahmadian, Mohammad Javan

**Affiliations:** ^1^ Institute of Biochemistry and Biophysics University of Tehran Tehran Iran; ^2^ Department of Physiology, School of Medical Sciences Tarbiat Modares University Tehran Iran; ^3^ Institute for Brain and Cognition Tarbiat Modares University Tehran Iran

**Keywords:** cellular aging, cuprizone, long‐term demyelination, multiple sclerosis, myelin repair impairment, oligodendrocyte progenitor cells, remyelination

## Abstract

Multiple sclerosis (MS) is a chronic inflammatory and demyelinating disorder affecting the central nervous system. Evidence suggests that age‐related neurodegeneration contributes to disability progression during the chronic stages of MS. Aging is characterized by decreased regeneration potential and impaired myelin repair in the brain. It is hypothesized that accelerated cellular aging contributes to the functional decline associated with neurodegenerative diseases. We assessed the impact of aging on myelin content in the corpus callosum (CC) and compared aging with the long‐term demyelination (LTD) consequents induced by 12 weeks of feeding with a cuprizone (CPZ) diet. Initially, evaluating myelin content in 2‐, 6‐, and 18‐month‐old mice revealed a reduction in myelin content, particularly at 18 months. Myelin thickness was decreased and the g‐ratio increased in aged mice. Although a lower myelin content and higher g‐ratio were observed in LTD model mice, compared to the normally aged mice, both aging and LTD exhibited relatively similar myelin ultrastructure. Our findings provide evidence that LTD exhibits the hallmarks of aging such as elevated expression of senescence‐associated genes, mitochondrial dysfunction, and high level of oxidative stress as observed following normal aging. We also investigated the senescence‐associated β‐galactosidase activity in O4^+^ late oligodendrocyte progenitor cells (OPCs). The senescent O4^+^/β‐galactosidase^+^ cells were elevated in the CPZ diet. Our data showed that the myelin degeneration in CC occurs throughout the lifespan, and LTD induced by CPZ accelerates the aging process which may explain the impairment of myelin repair in patients with progressive MS.

## Graphical abstract

We examined the effects of aging on myelin content in the corpus callosum (CC) and compared it with long‐term demyelination (LTD) induced by a 12‐week cuprizone diet. We investigated myelin changes, mitochondrial disruption, senescence occurrence, gliosis, and the aging of oligodendrocytes in the CC. Our findings revealed that myelin degeneration in the CC occurs throughout the lifespan, and CPZ might accelerate the aging process, which potentially leading to disruption in myelin repair.

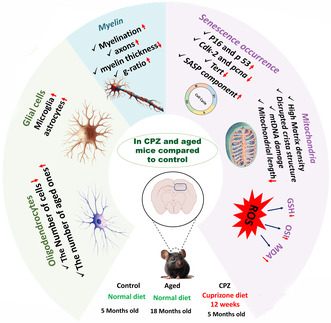

**Abbreviations**
CC: corpus callosum
CDK‐2: cyclin dependent kinase‐2
CNS: central nervous system
CPZ: cuprizone
GFAP: Glial fibrillary acidic protein
GSH: reduced glutathione
IBA1: ionized calcium‐binding adapter molecule 1
ICAM: intracellular adhesion molecule
IL‐6: intelukin‐6
LFB: luxol fast blue
LTD: long‐term demyelination
MBP: myelin basic protein
MDA: malondialdehyde
MS: multiple sclerosis
OPC: oligodendrocyte progenitor cells
OSI: oxidative stress indicator
PCNA: proliferating cell nuclear antigen
PLP: proteolipid protein
RRMS: relapsing‐remitting MS
SASP: senescence associated secretory phenotype
SA‐β‐gal: senescence‐associated β‐galactosidase
SPMS: secondary progressive MS
TEM: transmission electron microscopy
TGF‐β‐R: transforming growth factor beta receptor

## 1 INTRODUCTION

Multiple sclerosis (MS) is the most prevalent nontraumatic neuroinflammatory impairment in the young population (Dobson & Giovannoni, 2019). Prominent neuropathological characteristics of MS are inflammatory demyelinated lesions in the central nervous system (CNS) with gliosis (Dobson & Giovannoni, 2019) disruption of the blood–brain barrier along with oligodendrocytes death that may eventually lead to neuronal degeneration and axon loss (Absinta et al., 2019). There is evidence of myelin regeneration in the early stages of the disease; however, the efficiency of this process diminishes gradually as the disease progresses to a chronic state (Ruckh et al., 2012). While Some patients benefit from efficient repopulation of the lesions by oligodendrocyte progenitor cells (OPCs), this process is inadequate in a considerable percentage of MS patients (Boyd et al., 2013).

The course of the disease, which involves episodic relapsing and recovery in early stages, is known as relapsing–remitting MS (RRMS) and may enter into the progressive phase referred to as secondary progressive MS (SPMS) (Correale & Ysrraelit, 2022). The decline in remyelination efficacy occurs while a notable OPCs population in the lesion sites persists (Tepavčević & Lubetzki, 2022). Numerous studies have attempted to explain the observed myelin repair failure and OPC exhaustion. Microenvironment changes caused by inflammation and increased patient age are suggested as the main causes of this progression (Correale et al., 2017). In the experimental setting, the myelin repair failure happens following 12 weeks of feeding mice with a diet containing cuprizone (CPZ), a copper‐chelating agent. The CPZ model causes activation of glial cells, oligodendrocytes stress and apoptosis, demyelination, and neural dysfunction (Kipp et al., 2009).

Aging is an inescapable phenomenon characterized by a gradual decline of optimal physiological functioning, leading to the gradual debilitation of organisms (Kritsilis et al., 2018). The aging process is explained by the valid biomarkers introduced based on various aging mechanisms (Wagner et al., 2016). Mitochondrial dysfunction emerges as a critical player in producing reactive oxygen species (ROS), DNA damage, and accumulation of senescent cells representing some of the proposed mechanisms (Chinta et al., 2015). Normal brain aging is associated with an unsteady metabolic state, neuroinflammation, elevated senescence, neuronal loss, and myelin degeneration (Kritsilis et al., 2018). The myelin sheaths, which wrap around axons and provide efficient saltatory conductivity and metabolic support, gradually decline in function throughout adulthood, impacting the overall neural activity (Salzer & Zalc, 2016). As the individual age, the inability of OPCs to differentiate into mature oligodendrocytes, coupled with a decline in the myelinating capacity of differentiated oligodendrocytes (Tepavčević & Lubetzki, 2022) along with the neuroinflammation, leads to progressive loss of white matter and contribute in brain aging (Correale et al., 2017). This deterioration in myelination and the associated neuroinflammation may play a crucial role in the onset and progression of neurodegenerative diseases such as multiple sclerosis (Correale et al., 2017). There is evidence of the senescence of neural progenitor cells within the white matter lesions of progressive MS autopsy brain tissues (Nicaise et al., 2019). Accordingly, rejuvenating the aged neural progenitors/stem cells may restore the remyelination capacity (Neumann et al., 2019).

In this study, we hypothesized that possibly accelerated aging within the demyelinating lesions might contribute to the failure of myelin repair by exhausted OPCs. We compared myelin contents in mice of different ages as well as lesions with long‐term demyelination (LTD) to compare aspects of aging and prolonged myelin damage. We report similar alterations in myelin content, myelin sheaths ultrastructure, and mitochondrial dysfunction following aging and prolonged demyelination.

## 2 RESULT

### 2.1 Changes in myelination throughout normal aging

**FIGURE 1:**
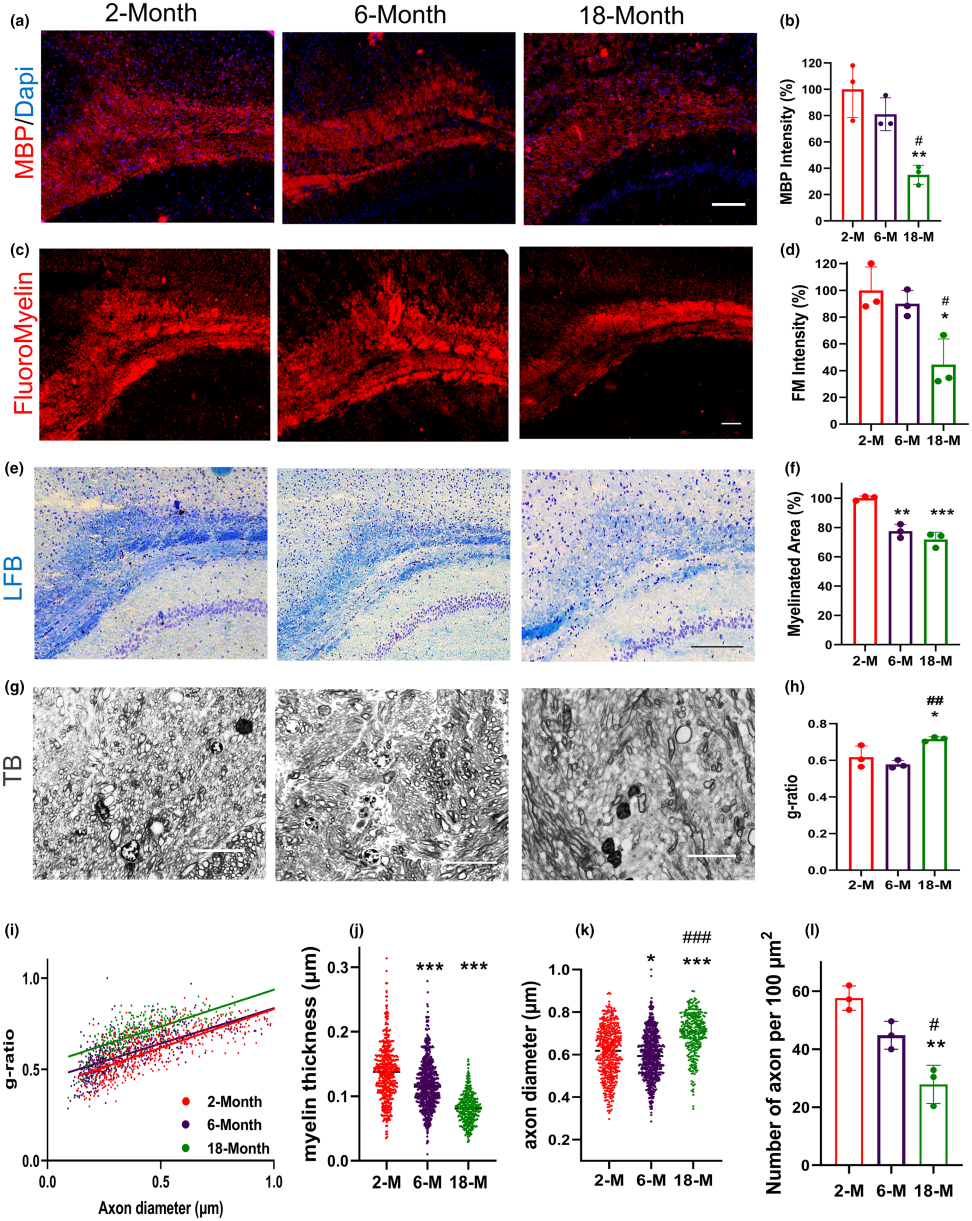
Myelination underwent changes throughout normal aging. (a) MBP immunofluorescence staining in the corpus callosa (CCs) of 2‐, 6‐, and 18‐month‐old mice. (b) Quantification of relative percentages of MBP intensity revealing a significant reduction in 18‐month‐old. (c) FluoroMyelin staining in the CCs of 2‐, 6‐, and 18‐month‐old mice, (d) the relative percentage of Fluoromyelin intensity decreased significantly in 18‐month‐old mice. (e) LFB staining in the CCs of 2‐, 6‐, and 18‐month‐old mice. (f) Quantification of relative percentage of myelinated area in LFB staining showed a significant reduction in the CCs of 6‐, and 18‐month‐old mice compared to 2‐month‐old mice. (g) Representative images of toluidine blue staining of semithin sections of CCs in 2‐, 6‐, and 18‐month‐old mice, scale bar 10 μm. (h) The g‐ratio analysis in 2‐, 6‐, and 18‐month‐old mice, the bars represent the median of the g‐ratio per individual. The g‐ratio was calculated using ImageJ software, measuring the inner axonal diameter divided by the diameter of the myelinated axon measured in every grided section, for each mouse three different sections of the CC were analyzed. (i) Scatter‐graph showing g‐ratio per axon diameter, representing an increase in g‐ratio with aging, (j) column scatter plot of myelin diameter changes showing alteration in myelin diameter during lifetime, (k) column scatter plot of axon diameter changes, and (l) the number of axons per 100 μm^2^ were measured in CCs of 2‐, 6‐, and 18‐month‐old mice. The scale bar is 100 μm in a, c, e, and 10 μm in g. Statistical analyses included one‐way ANOVA and Tukey multiple comparison tests for b, d, f, h, and l and Kruskal‐Wallis tests for j and k. All experiments were conducted in three replicates, with *n* = 3 mice per group. *Represent comparison between 2‐month‐old and other groups and # represent a comparison between 6‐month‐old group and 18‐month‐old. * or #*p* < 0.05, ** or ##*p* = 0.001, *** or ###*p* < 0.001 between conditions.

To characterize changes in the myelination levels during the lifespan, we evaluated myelination intensity in the CC using anti‐MBP, FluoroMyelin, and luxol fast blue (LFB) staining. As mentioned in Figure 1a, b, MBP expression, the main protein of myelin sheaths, dropped to 35.07% in 18‐month‐old mice which is significantly lower compared to 2‐ (*p* = 0.004) and 6‐month‐old mice (*p* = 0.022). Myelin content was also evaluated by FluoroMyelin staining, which showed the same pattern of reduction in myelination over time (Figure 1c,d). The FluoroMyelin staining intensity remained relatively stable during reproductive ages and reduced to 44.48% in 18‐month‐old mice compared to 2‐ (*p* = 0.02) and 18‐month‐old mice (*p* = 0.04). Figure 1e showed the extent of the area stained with LFB within the CC. Myelination area reduced to 77.49% (*p* = 0.011) in 6‐month‐old mice and dropped to 71.84% (*p* = 0.003) in 18‐month‐old mice (Figure 1f). Our results indicated myelin content declined over time.

To enhance our understanding of the variations in white matter throughout the lifespan, we stained the semithin sections of the CC with toluidine blue and compared the groups. Sample micrographs are presented in Figure 1g. Then, we analyzed the micrographs for g‐ratio, myelin sheath, axon diameter thickness, and the number of axons. The g‐ratio median calculated for each mouse remained consistent between 2‐month‐old and 6‐month‐old mice; however, a significant increase was observed in 18‐month‐old mice compared to 2‐ (*p* = 0.04) and 6‐month‐old (*p* = 0.009) groups (Figure 1h). Similar findings were noticed in the analysis of g‐ratio against axon diameter (Figure 1i). The g‐ratio plot in 2‐ and 6‐month‐old mice remained unchanged, while an increase was observed in 18‐month‐old mice g‐ratio. Myelin sheath thickness declined (*p* < 0.001) in 18‐month‐old mice compared to both 2‐ and 6‐month‐old mice (Figure 1j). The evaluation of axon diameter variation exhibited a reduction from 2‐month‐old mice to 6‐month‐old mice (*p* = 0.02), and a considerable elevation (*p* < 0.001) in 18‐month‐old compared to both 2‐ and 6‐month‐old mice (Figure 1k). Additionally, we assessed, whether the axonal count has been altered during the lifetime. The abundance of axons did not reveal a significant reduction in 6‐month‐old mice compared to 2‐month‐old mice; however, it significantly declined in 18‐month‐old mice (*p* < 0.001) compared to both 2‐ and 6‐month‐old mice (Figure 1l). These findings suggest diminished myelin content, myelin unsheathing, and eventually axonal loss throughout the lifetime.

### 2.2 Comparing changes in myelination between aged and cuprizone demyelinated mice

**FIGURE 2:**
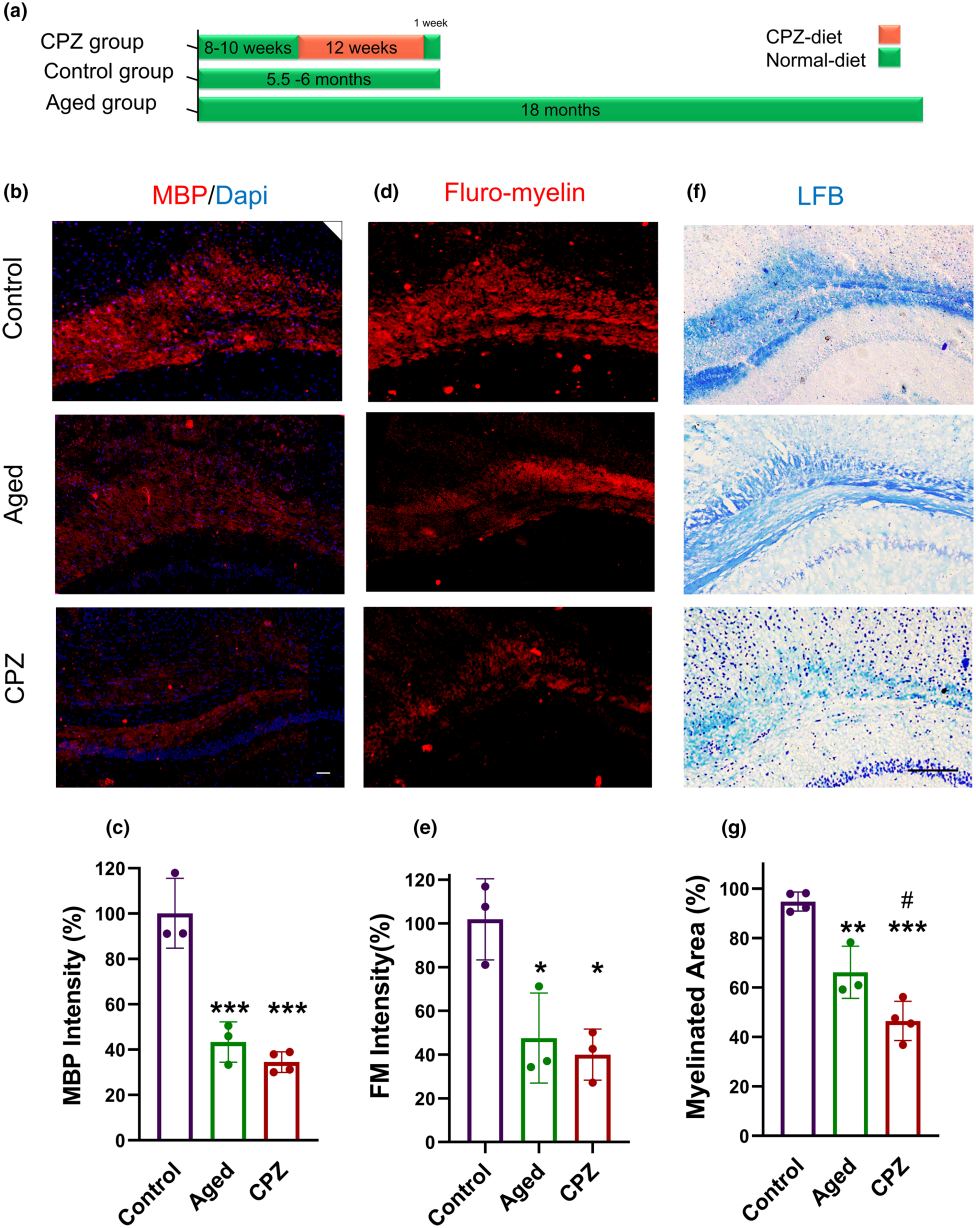
Cuprizone‐fed mice represent a myelin loss pattern similar to aged mice. (a) A timeline illustrating the experimental design of the study. (b) MBP immunofluorescence images and (c) quantification of relative percentages of MBP intensity in the corpus callosa (CCs) of Control, Aged, and CPZ mice. (d) LFB staining images and (e) Relative myelinated area percent in the CCs of Control, Aged, and CPZ mice based on LFB staining. (f) FluoroMyelin staining images and (g) relative percentages of FluoroMyelin intensity in the CCs of Control, Aged, and CPZ mice. scale bar is 50 μm in b and 100 μm in d, and f. Statistical analyses were performed using one‐way ANOVA and Tukey multiple comparison tests. All experiments were conducted in three replicates, with *n* = 3 mice per group. *Represent comparison between Control and other groups and # represent comparison between Aged group and CPZ. * or #*p* < 0.03 **p < 0.003; ***p < 0.001.

Demyelination has originally been identified as a hallmark of MS disease, and LTD leads to remyelination failure. In this study, we aimed to examine whether myelination impairment associated with LTD is comparable to the changes observed during aging. We used a 12‐week CPZ diet as the LTD model and compared the animals with the same‐age control mice (~6 months) on a normal diet (Control) and 18‐month‐old mice undergoing normal aging (Aged, Figure 2a).

**FIGURE 3:**
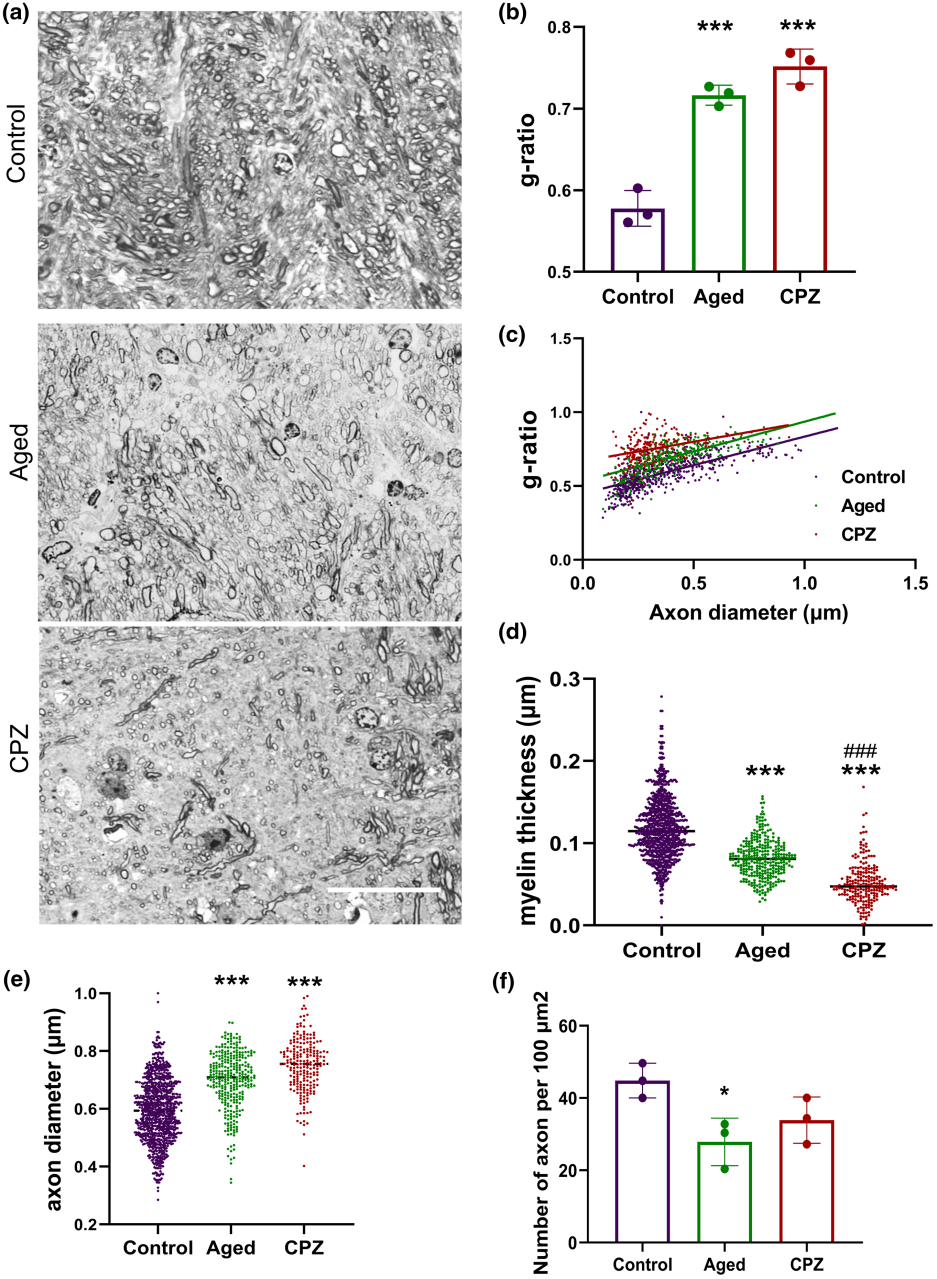
The ensheathment of myelin was affected by both aging and LTD similarly. (a) representative images of toluidine blue staining of semithin sections of the corpus callosum (CC) in Control, CPZ, and Aged mice. (b) the median g‐ratio median per individual was quantified in the Control, Aged, and CPZ groups. (c) The g‐ratio per axon diameter scatter plot, each dot represents one measurement of g‐ratio per its axon diameter. (d) Column scatter plot of axon diameter changes (e) column scatter plot of myelin thickness changes and (f) the number of axons per 100 μm^2^ was measured in CC of Control, Aged, and CPZ mice. The scale bar is 10 μm in a. Statistical analyses included one‐way ANOVA and Tukey multiple comparison tests for b and f, and Kruskal‐Wallis tests for d and e. All experiments were conducted in three replicates, with *n* = 3 mice per group. *Represent comparison between Control and other groups and # represent comparison between Aged group and CPZ *p = 0.03, *** or ###*p* < 0.001.

We evaluated MBP expression in the described groups in CC (Figure 2b). MBP intensity was reduced in both normal aging (44.32%, *p* < 0.001) and CPZ (34.52% *p* < 0.001) groups (Figure 2c). Although the MBP intensity in the CPZ group was approximately 8.77% lower than in the Aged group, the difference was not significant (*p* = 0.52). FluoroMyelin and LFB staining also confirmed a reduction in myelin content in the Aged and CPZ groups compared to the Control group (Figure 2d, f). Quantitative data revealed a significant reduction in FluoroMyelin intensity 47.56% (*p* = 0.02) and 40.00% (*p* = 0.01), respectively, in CPZ and Aged mice (Figure 3e). The myelinated area in LFB staining was reduced in Aged mice by 28.60% (*p* = 0.003) and in CPZ mice by 48.24% (*p* < 0.001, Figure 2f).

Furthermore, the analysis of semithin sections of the CC stained with toluidine blue (Figure 3a) revealed a significant elevation in the g‐ratio median per mice within the Aged and CPZ groups (both, *p* < 0.001) compared to the Control group (Figure 3b). The profiling of the g‐ratio against axon diameter revealed similar results with elevated g‐ratio in the Aged and CPZ group (Figure 3c). Figure 3d shows that myelin sheath reduced substantially within both Aged and CPZ groups (*p* < 0.001), while the axon diameter was expanded significantly in these groups (both, *p* < 0.001, Figure 3e). To evaluate the effect of aging and chronic demyelination on axonal loss we also measured changes in the number of axons. The number of axons declined significantly only in the Aged group (*p* = 0.03), while a decrease in this parameter in CPZ mice was not significant (Figure 3f). These findings suggest a substantial reduction in myelination in both Aged and CPZ mice compared to Control, while there were minor distinctions between these two groups.

### 2.3 Changes in myelin ultrastructure in aging and CPZ‐demyelination

**FIGURE 4:**
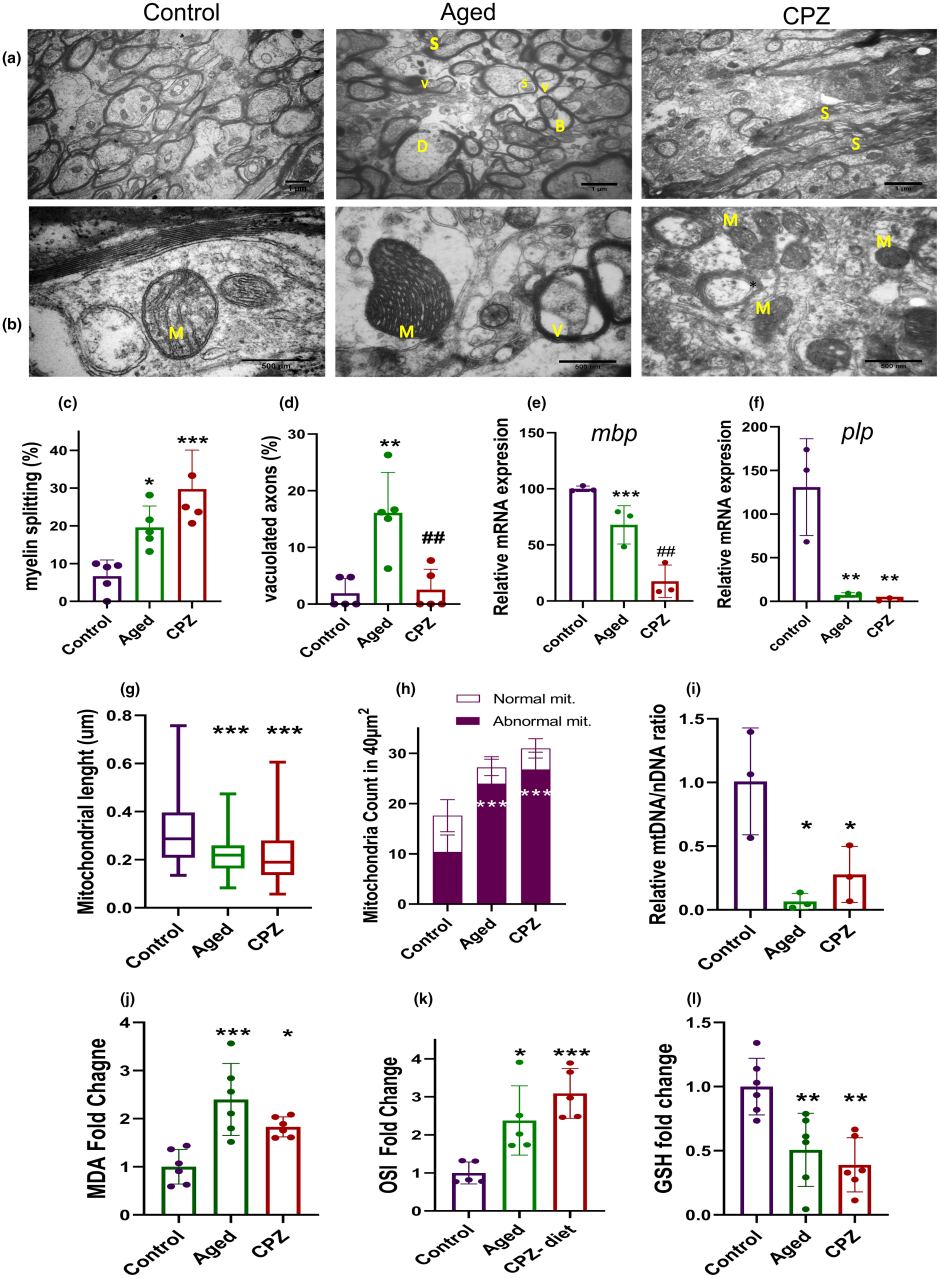
Aging and CPZ revealed distinct ultrastructure features within the corpus callosum CC. (a and b) Representative transmission electron microscopy (TEM) images of the corpus callosum (CC) from the Control, Aged, and CPZ groups, showing myelin splitting (indicated by S), myelin balloon formation (indicated by B), myelin vacuoles formation (indicated by V) and ultrastructural variation of mitochondria (indicated by M). (c) The percentage of myelin splitting, (d) vacuolated axons percentage in TEM images of Control, Aged, and CPZ mice. (e and f) Relative mRNA level of *mbp* and *plp* in the CCs were measured in Control, Aged and, CPZ mice. (g) Alterations in mitochondrial length were assessed in the Control, Aged, and CPZ groups. (h) Quantification of the total number of mitochondria in 40 μm^2^ of TEM images was performed in the Control, Aged, and CPZ groups, distinguishing normal (normal matrix, parallel, and arranged crista) and abnormal mitochondria (dense matrix, disrupted membrane, and crista), as specified in the graph. (i) The relative ratio of mitochondrial DNA copy number to nuclear DNA copy number, revealed a reduction by Aging and CPZ. (j) the fold change in the malondialdehyde (MDA) levels, (k) oxidative stress indicator (OSI) measurements, and (l) the fold change in reduced GSH levels were analyzed in Control, Aged, and CPZ mice. The scale bar is 500 nm. Statistical analyses included one‐way ANOVA and Tukey multiple comparison tests for c‐f and j‐l, Kruskal‐Wallis tests for g, and two‐way ANOVA and Tukey multiple comparison tests for h. All experiments were conducted in three replicates each. Sections a‐d and g‐I were triplicated. In sections e‐f n = 6 mice per group were used (two mice CCs were pooled together as one sample) and in sections j‐l 4–5 mice per group were included. * Represents a comparison between Control and other groups, and # represents a comparison between the Aged group and CPZ. *p < 0.05, ** or ##*p* < 0.01, ***p < 0.001.

We performed an ultrastructural study on CC sections from Control, Aged, and CPZ groups using transmission electron microscopy (TEM). As shown in Figure 4a,b, the evaluation of CC in Control mice revealed consistent myelinated axons with unified and compact myelin sheaths. However, both Aged and CPZ mice exhibited ultrastructural alterations in myelin structure. We observed localized degeneration of myelin sheaths in some axons (indicated by D in Figure 4a) and splitting between compact myelin sheaths (indicated by S in Figure 4a). This splitting could lead to the bulging out of cytoplasm, known as myelin balloon (indicated by B in Figure 4a) and, in some cases, the formation of vacuole‐like structures around the axons (indicated by V in Figure 4a,b).

While there were similarities in the ultrastructural features of both Aged and CPZ groups, the analysis of TEM sections for these two groups revealed some distinctions. The splitting of myelin lamellae in the Aged mice occurred only in certain regions surrounding the axon. In contrast, in the CPZ mice, this splitting was more extensive, involving a larger portion of the axons. As illustrated in Figure 4c, the splitting of the compact myelin sheath around the axons was (19.6%, *p* = 0.04) in Aged and (29.7%, *p* < 0.001) in CPZ diet mice. The quantification of the abundance of vacuolated axons showed a significantly higher number of vacuolated axons in the Aged group (21.02%, *p* < 0.001) and compared to CPZ mice (8.54%, *p* = 0.05). The number of vacuolated axons was higher in Aged mice (Figure 4d). Since compact multilayered myelin structure are disrupted in aged and CPZ mice, we investigated the expression of myelin basic protein (MBP) and proteolipid protein (PLP) genes, which are critical for the formation of the compact myelin structures (Figure 4e,f). *Mbp* expression decreased significantly in CPZ group (82.47%, *p* < 0.001, Figure 4e). While there was a reduction in *mbp* expression in aged group (32.14%), this reduction was not statistically significant. The expression *plp* reduced in both aged (7.26%, *p* = 0.007) and CPZ (2.22%, *p* = 0.006) groups compared to Control group (Figure 4f). Additionally, the decreased level of MBP was mentioned in Figure 2b,c.

Mitochondrial dysfunction is a major driving force in aging (Elfawy & Das, 2019; Trifunovic & Larsson, 2008). We evaluated mitochondrial features in the brain sections and the oxidative stress status in brain homogenates following aging and prolonged CPZ‐demyelination. The ultrastructural examination of CC exhibited a significant difference in the mitochondrial ultrastructure among Control, Aged, and CPZ mice, which is indicated by M (Figure 4b). In CC sections of the Control mice, mitochondria displayed a uniform appearance with distinct cristae, arranged in parallel shapes, well preserved surrounding membranes with normal matrix density. In contrast, mitochondria in Aged mice and CPZ exhibited variations in the internal organization, including some disrupted cristae structures, interconnected inner boundary membranes, irregular junctions between cristae, high matrix density, as well as the formation of vacuole‐like structures particularly observed in CPZ mice. We measured the length of mitochondria in different sections (Figure 4g). While the mitochondria of the Control group were the smallest, they were swollen in both Aged and CPZ mice (both *p* < 0.001). We also compared the mitochondria count and the percentage of normal and abnormal mitochondria in Aged and CPZ mice. Data revealed a total increase in the mitochondria abundance in the Aged and CPZ group (Figure 4h). While there was not a significant reduction in number of normal mitochondria, the number of mitochondria with abnormal structures was increased in the Aged by 28.49% (*p* < 0.001) and CPZ 26.79% (*p* < 0.001) groups (Figure 4h).

Since there were obvious ultrastructural alterations in mitochondria appearance and abundance, we assessed the mtDNA/nDNA ratio as an indicator of mitochondrial abnormality, which is associated with aging (Dolcini et al., 2020) (Figure 4i). A reduction in mtDNA/nDNA ratio suggests mtDNA damage in both Aged and CPZ mice. Additionally, oxidative stress indicator was measured in brain homogenates, including malondialdehyde (MDA), oxidative stress indicator (OSI), and reduced glutathione (GSH) levels, shown in Figure 4j–l, respectively. There were significantly higher oxidative stress indicators in both Aged and CPZ mice compared to Control. The MDA level was elevated by 2.39‐fold (*p* < 0.001) in Aged and 1.83‐fold (*p* = 0.027) in CPZ mice (Figure 4j). The OSI increased 2.38‐fold (*p* = 0.02) in Aged and 3.09‐fold (*p* < 0.001) in CPZ mice (Figure 4k). The evaluation of reduced GSH exhibited a reduction by 0.506‐fold change (*p* = 0.008) in the Aged and a 0.039‐fold change (*p* < 0.001) in the CPZ diet (Figure 4l).

### 2.4 Changes in senescence‐associated factors and tissue gliosis

**FIGURE 5:**
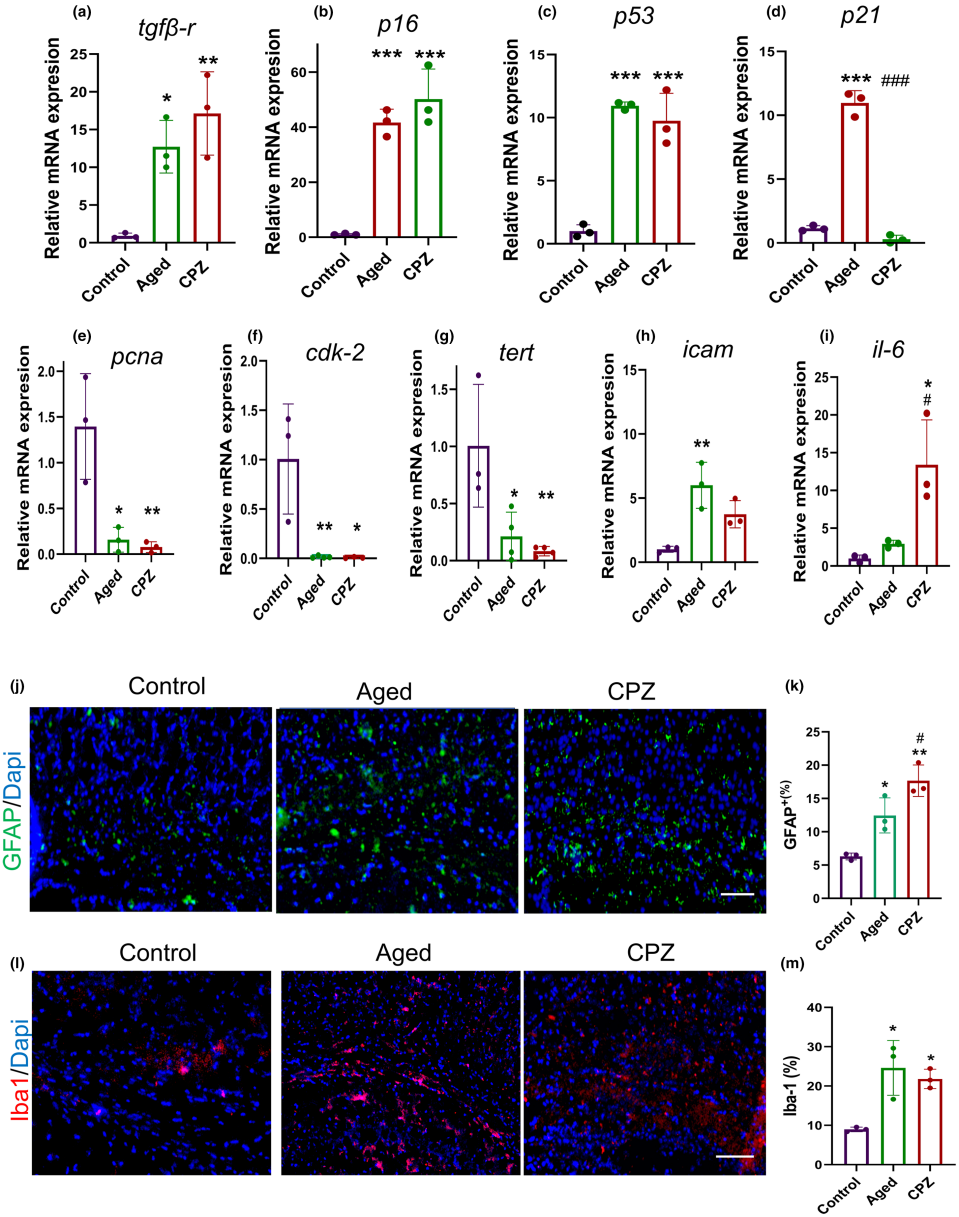
Cuprizone modulates the expression of genes associated with senescence and SASP. The relative mRNA levels of (a) *tgf‐βr*, (b) *p16*, (c) *p53*, (d) *p21*, (e) *pcna*, (f) *cdk‐2*, (g) *tert*, (h) *icam*, and (i) *il‐6* in the corpus callosum (CC) were measured in Control, Aged and, CPZ mice. (j) Immunofluorescence staining of astrocytes in Control, Aged, and CPZ mice CCs. (k) Quantification of the percentage of the GFAP^+^ cells. (l) Immunofluorescence staining of microglia. (m) Quantification of the percentage of the IBA‐1^+^ cells in Control, Aged, and CPZ mice CCs. The scale bar is 20 μm. Statistical analysis was performed using one‐way ANOVA and Tukey multiple comparison tests. All experiments were conducted in three replicates. In a–i there were *n* = 6 mice per group (two mice CCs were pooled together as one sample), in j‐m three mice were assigned to each group. * represents the comparison between Control and other groups and # represents a comparison between the Aged group and CPZ. * or #*p* < 0.025, **p < 0.01 and, *** or ###*p* < 0.001.

Aging is associated with cellular damage, leading to cellular senescence and permanent cell cycle arrest (Ogrodnik, 2021). We attempted to identify how LTD and aging affect the cell cycle. We assessed the expression of cell cycle inhibitor genes *tgf‐br*, *p53*, *p16* ^*ink4a*^, and *p21* and the cell cycle promoter genes *cdk‐2*, *pcna*, and *tert*. *Tgf‐br*, *p53*, and *p16* ^*ink4a*^ revealed increased expression in both Aged (12.73‐fold, *p* = 0.02), (10.94‐fold, *p* < 0.001), and (41.66, *p* < 0.001) and CPZ mice (17.13, *p* = 0.004), (9.96‐fold, *p* < 0.001), and (50.22‐fold, *p* < 0.001), respectively, compared to Control (Figure 5a–c). The expression of *p21* showed significant elevation only in Aged mice (10.96‐fold, *p* < 0.001) with no significant change in CPZ mice (Figure 5D). The expression level of *pcna*, *Cdk‐2*, and *tert* displayed the same pattern. It significantly decreased in Aged (0.15‐fold, *p* = 0.01), (0.01‐fold, *p* = 0.008), (0.21‐fold, *p* = 0.02), and CPZ mice (0.07‐fold, *p* = 0.008), (0.008‐fold, *p* = 0.01), and (0.08‐fold, *p* = 0.009), respectively (Figure 5e–g).

We further investigated the expression levels of pro‐inflammatory factors acting as senescence‐associated secretory phenotype (SASP) components. *Icam* expression increased 6.00‐fold (*p* = 0.006) in Aged mice compared to Control (Figure 5h). Although the level of *icam* showed an increasing trend in the CPZ diet (3.74‐fold), its alteration was insignificant (*p* = 0.7). Data also indicated a 13.41‐fold upregulation of *il‐6* expression level in the CPZ group (*p* = 0.01, Figure 5i). These results suggest that the expression of genes related to the progression of the cell cycle was dropped, and the expression of genes responsible for cell cycle arrests was almost increased in both Aged and CPZ mice.

Additionally, we assessed the possible astrogliosis and microgliosis in CC following aging and LTD. GFAP immunostaining was used to identify astrocytes in Aging, CPZ, and Control groups (Figure 5j). GFAP^+^ cells were increased 1.97‐fold (*p* < 0.02) in Aged and 2.80‐fold (*p* < 0.001) in CPZ compared to Control (Figure 5k). We also determined alteration of microglial and resident macrophages cells by immunostaining (Figure 5l). Immunostaining revealed an increase in the population of Iba‐1^+^ in both Aged (2.74‐fold, *p* < 0.01) and CPZ (2.43‐fold, *p* < 0.02) mice compared to Control.

### 2.5 Oligodendrocytes undergo accelerated aging following long‐term demyelination

**FIGURE 6:**
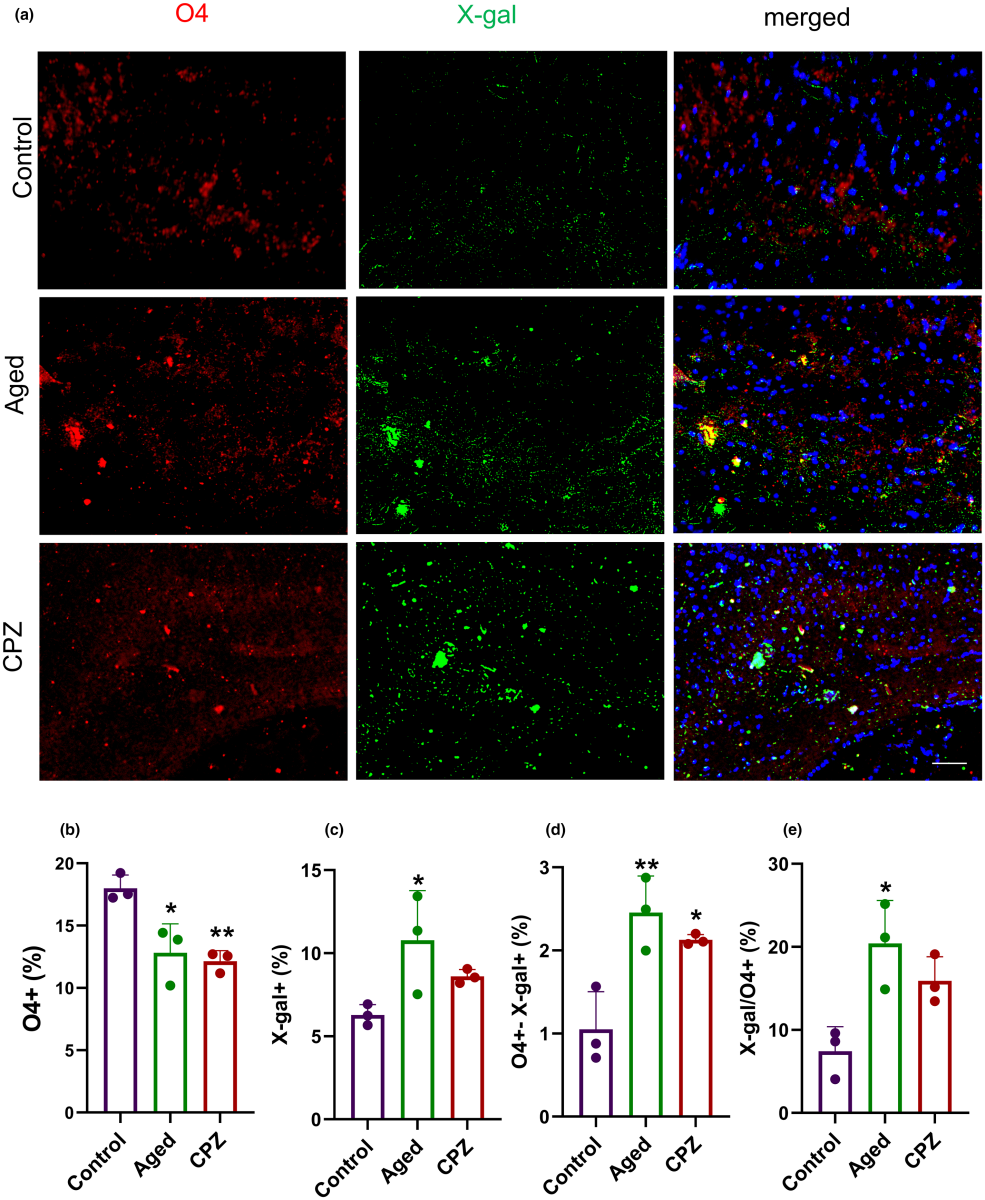
The O4 oligodendrocyte population decreased in the cuprizone diet with an elevation in the activity of senescence‐associated with β‐galactosidase (SA‐β gal) activity. (a) Immunofluorescence staining O4 late OPCs and SA‐β‐gal staining as a marker of cell cycle arrest in Control, Aged, and CPZ mice in the corpus callosum (CC) revealed a decrease in the percentage of late OPC during aging and CPZ beside an increase in SA‐β‐gal. (b) Quantification of the percentage of the O4^+^ cells. (c) Quantification of the percentage of SA‐ β‐gal^+^ cells. (d) The percentage of cells that are O4^+^ also shows SA‐ β‐gal activity. (e) The percentage of cells showing SA‐ β‐gal activity per O4^+^ cells. The scale bar is 20 μm. Statistical analyses were performed using one‐way ANOVA and Tukey multiple comparison tests. All experiments were conducted in three replicates, with *n* = 3 mice per group. *Represent comparison between Control and other groups. *p < 0.05, **p < 0.01.

Since reduced myelin content and demyelination, as well as aging biomarkers, were observed following both normal aging and CPZ‐induced prolonged demyelination, we hypothesized that LTD might lead to OPCs dysfunction through the aging occurrence. We evaluated the presence of the $O4^{+}$ late OPCs in CC through immunofluorescence staining. Additionally, we co‐stained the sections to determine the presence of senescence‐associated β‐galactosidase (SA‐β‐gal) activity, a marker of cellular senescence (Figure 6a). Immunostaining revealed a substantial depletion of $O4^{+}$ cells in Aged (5.16% reduction, *p* = 0.02) and CPZ mice (5.85% reduction, *p* = 0.008) compared to Control (Figure 6b). The percentage of SA‐β‐gal cells was significantly elevated in the Aged (4.49% increase, *p* = 0.04) and showed a non‐significant elevation in CPZ (2.32% increase, *p* = 0.3) groups (Figure 6c). Consistent with this, compared to Control, the accumulation of senescent late OPCs was detected by the presence of O_4_ and SA‐β‐gal positive cells in the Aged (1.40%, *p* = 0.008) and the CPZ mice (1.07%, *p* = 0.03, Figure 6d). The relative percentage of the X‐gal positive cells to the O_4_ cell also increased significantly in Aged mice (19.97%, *p* = 0.01, Figure 6e). Aged and CPZ OPCs exhibited a substantial increase in the activity of SA‐β‐Gal, which indicates the occurrence of cell senescence and growth arrest following aging and long‐term demyelination.

## 3 DISCUSSION

This study initially aimed to elucidate alterations in the myelin sheath throughout the mice's life span. Myelin sheaths are relatively stable once formed and degenerate during aging (Absinta et al., 2020). Our data showed a reduction in myelin content throughout the lifespan, especially in advanced ages (Figure 1), a significant decline in myelin content was observed in 18‐month‐old mice. A previous report also suggested reduced myelin content in the cortex of mice of the same age (Wang et al., 2020). Aging led to a significant increase in axon diameter and a decrease in myelin thickness that manifested as the elevation of the g‐ratio. The g‐ratio is associated with the speed of action potential conduction and thus reflects the axon's functional integrity (Ikeda & Oka, 2012). These findings aligned with the earlier reports indicating g‐ratio alterations in the white matter associated with aging (Xie et al., 2014). We also evaluated the axon abundance in CC, which revealed a 51% reduction in aged mice compared to 2‐month‐old mice. Age‐related axonal loss has been observed in various parts of the nervous system in humans and experimental animals (Marner et al., 2003). White matter loss exceeds in many neurodegenerative diseases in the elderly (Nasrabady et al., 2018).

We evaluated the difference in myelin loss between normal aging and chronic demyelination induced by CPZ. The 12 weeks‐CPZ‐fed mice, a model of the prolonged demyelination, were compared to 6‐month‐ and 18‐month‐old mice. Both aged and CPZ mice exhibited the same reduction pattern in myelin content. MBP expression and increased g‐ratio within CC fibers confirmed a decrease in myelin content in both aging and CPZ diet mice.

Electron microscopy studies have established the changes in myelinated nerve fiber morphology as the major alteration observed during normal aging (Stadelmann et al., 2019). Our results demonstrated an increased prevalence of abnormal myelin appearance, such as myelin lamellae splitting, vacuolar structure formation, presence of myelin balloons, myelin degeneration, and lower myelin content in both aged and CPZ mice. These results underline similarities between normal aging and the prolonged demyelinated model. This provides evidence for the aging‐related ultrastructure changes impairing CC integrity and axonal conduction velocity during normal aging (Xie et al., 2014). The same changes were reported in the CPZ model of demyelination (Joost et al., 2022). Here, some ultrastructural characteristics, such as the splitting of the myelin lamellae, were more pronounced in the CPZ mice. In contrast, others, such as the formation of vacuoles and balloons, were more prominent in normal aging. PLP and MBP are both important to provide compact structure of myelin. Our study revealed reduce expression of these genes in both Aged and CPZ which might imply for the myelin splitting.

Among aging theories, the mitochondrial free radical theory of aging has been important for several decades. According to this theory, ROS are toxic byproducts of aerobic metabolism that cause oxidative damage to various cellular components. Accumulating evidence has suggested a pivotal connection between mitochondrial dysfunction and the major phenotypes associated with aging (Jang et al., 2018). The morphological changes in the mitochondria, such as packed lamellar cristae resulting in a spiral‐like cristae network, and the dense matrices are important aging features (Jiang et al., 2017). We detected this abnormal structure of mitochondria in aged‐ and CPZ‐mice. Accumulation of mtDNA mutations and reduced mitochondrial DNA copy number (mtDNA‐CN) is a biomarker of mitochondrial dysfunction in aging and various chronic diseases (Montier et al., 2009). In a cellular model, mtDNA depletion caused reduced expression of vital complex proteins, altered cellular morphology, and lowered respiratory enzyme activity (Jeng et al., 2008). Our results demonstrated a lower mtDNA‐CN in both aging and CPZ demyelination that potentially contribute to the mitochondrial defects and aging characteristic in 18‐month‐old mice as well as CPZ. Increased levels of oxidative phosphorylation due to impaired mitochondrial function and morphology are known as another hallmark of aging (Bratic & Larsson, 2013). Increased biomarkers of ROS such as MDA, reduced‐GSH, and OSI in aged and CPZ diet mice reflected the elevated oxidative stress.

Previous studies have well established that cell senescence potentially contributes as a substantial factor in the progress of aging in mammals (Kumari & Jat, 2021). This might be due to the act of senescent cells as a continuous source of cell cycle arrest stimuli and pro‐inflammatory agents that lead to the loss of organs' homeostasis and function (Jurk et al., 2012). Our results demonstrated the downregulation of *cdk‐2*, *pcna*, and *tert*, which restrain cell cycle progression in normal aging and CPZ models. Downregulation of *tert* causes telomer shortening, which correlates with impaired tissue injury response and myelination decline (Jaskelioff et al., 2011). Reports indicate that telomere length is inversely associated with disability in MS patients (Bühring et al., 2021). Here, TGF‐β, a key regulator in the brain of aged mice, was upregulated in CC of the Aged and CPZ mice. TGF‐β provokes cellular senescence, increases cyclin‐dependent kinase inhibitors p16 and p53, and causes telomer shortening and upregulation of SASP (Bernhardi Montgomery et al., 2015). P53, which induces senescence and is considered as a hallmark of cellular aging, was elevated in both aged and CPZ mice. The measurement of the p16 expression, as a protein associated with permanent cell cycle arrest (Serra & Chetty, 2018), revealed a similar pattern and showed elevation in both aged and LTD. The increase in p16 besides p53, provides a secondary barrier to restrain cell cycle progression. P21 expression which is directly activated by the p53 protein, suppresses CDKs and leads to G1‐S cell cycle arrest (Engeland, 2022). In our study, *p21* was elevated only in aged mice indicating its role in cell cycle arrest during mice aging. It may not contribute to LTD‐induced cell aging phenotypes. Additionally, we measured the expression of *Icam* and *Il‐6* genes as SASP components. The expression of *il‐6* was significantly higher only in LTD conditions. It is also reported that IL‐6 may contribute to the occurrence of premature senescence in human fibroblasts (Kojima et al., 2012), and its overexpression mediates oncogene‐induced senescence (Kuilman et al., 2008). IL‐6 is known as a cerebrospinal fluid biomarker of MS and its expression has been associated with the disability progress in patients with MS (Stampanoni Bassi et al., 2020). Icam is another SASP factors that interfere with blood–brain barrier integrity during aging and lead to neuroinflammation (Graves & Baker, 2020). In our study, its expression was increased only in Aged mice. Changes in evaluated genes support the cellular senescence and decreased regeneration capacity observed in the brains of Aged and CPZ mice.

Microglia and astrocytes are key players of inflammatory responses in the central nervous system (Kwon & Koh, 2020). The increased inflammation mediated by microglia and astrocytes are known as a driving force in aging and neurodegenerative diseases (Pan et al., 2020). Here, we observed the presence of astrogliosis and microgliosis in both Aged and CPZ groups. Therefore, the increased level of inflammatory factors might be the direct outcome of gliosis, as well as the changes in oligodendrocytes secretome due to senescence.

Oligodendroglia lineage cells are among the main populations of cells in CC. It was reported that senescence could disrupt OPCs differentiation and proliferation, besides the functional impairment (Zhang et al., 2019). It has been previously confirmed that the cellular senescence of progenitor cells diminishes remyelination in progressive MS (Nicaise et al., 2019). Our results represented the elevation of SA‐β‐gal positive cells in CC of Aged and LTD mice, demonstrating senescence incidence. We confirmed that the percentage of O4^+^ late OPCs was decreased in CC along with the elevation of the percentage of O4^+^ cells that express SA‐β‐gal in LTD and Aged mice. Therefore, OPCs senescence seems as a mechanism of OPC maturation failure following LTD in animals with 12 weeks of CPZ diet.

In this study, we observed a significant reduction in myelin content in Aged and LTD mice. Then we provided structural and molecular comparison between LTD and Aged mice revealing that the extent of the myelin reduction during healthy brain aging was similar to the myelin content reduction observed in LTD mice. Our results exhibited that the age‐related elevation of SA‐β‐gal positive cells in CC was accompanied by escalated senescence‐associated genes, mitochondrial dysfunction, and an increase in oxidative stress. This premature aging phenotype may explain the exhaustion of OPCs following 12 weeks of CPZ treatment and failure of myelin repair in patients suffering from progressive MS. Accordingly, antiaging treatments and cellular aging reversal treatments may promise tools to manage the MS disease course.

## 4 METHOD AND MATERIAL

### 4.1 Animals

Male C57BL6/J mice were purchased from the Pasteur Institute of Iran (Karaj, Iran). Animal procedures and handling were done according to the ARRIVE guidelines for working with laboratory animals. Mice were kept in standard cages with 12‐h light/dark cycle and room temperature of 23 ± 2°C. Animals were fed with standard rodent chow and accessed water Ad libitum. All experimental protocols were approved by the Committee for Ethics in Animal Research of Tehran University Faculty of Sciences, Teran, Iran (Approval ID: IR.UT.SCIENCE.REC.1400.014). Attempts were conducted to minimize the animals' suffering and reduce the number of animals used.

### 4.2 Cuprizone‐induced demyelination

CPZ is a cooper chelating agent that provides a pathology like MS lesions following oral administration in animal chow (Kipp et al., 2009). The rodent chow was minced and supplemented with 0.2% CPZ (Sigma‐Aldrich, #Cas 370–81‐0). To ensure the effectiveness of the CPZ pellet, we prepared them freshly and kept them for less than 3 days. 8‐week‐old mice were fed with the CPZ‐supplemented pellet for 12 weeks to induce chronic demyelination, which leads to OPCs exhaustion. Animals returned to normal chow after 12 weeks and were kept for another week before being sacrificed.

### 4.3 Experiment design

To administer long‐term demyelination, 6‐week‐old mice were purchased and kept for 2 weeks to acclimate. Mice were randomly assigned to the CPZ or Control groups. The CPZ group was fed with CPZ‐supplemented chow. Control group was fed with normal chow. For the aged animal group, we bought 8‐month‐old mice and kept them in the animal facility until they reached 18 months. All mice were randomly assigned to different subgroups.

### 4.4 Tissue preparation

For tissue sampling, mice were anesthetized deeply with an intraperitoneal injection of ketamine (Bremer Pharma GmbH) and xylazine (Kela), then perfused transcardially with 0.1 M phosphate buffer saline (PBS). Tissue preparation was performed according to the Cold Spring Harbor Laboratory Cryo sectioning Tissues protocol. The brains were harvested, frozen in liquid nitrogen, immediately embedded in optimal cutting temperature (OCT, Bio‐Optica) compound, and cryo‐sectioned (Histo‐Line Laboratories) at 10 μm. All sections were preserved at −70°C till use.

### 4.5 Immunostaining

Cryosections of three mice were washed with 0.1 M PBS and fixed using 4% paraformaldehyde (PFA) for 10 min. After fixation, they were permeabilized using Triton X‐100 (0.3%) and blocked with 10% normal goat serum and 0.2% Triton X‐100 prepared in 0.1 M PBS for 1 h at room temperature. Sections were then stained using the following primary antibodies overnight at 4°C. The primary antibodies used in this study were anti‐MBP (1:1000, Aves Labs Cat# MBP‐0020), O4 (1:250, Merk, Cat# MAP345M), GFAP (1:500, Dako, Cat#Z0334) and Iba1 (1:200, santa cruz, Cat#sc98468). After incubation with primary antibody, sections were washed with PBS, and incubated with appropriate secondary antibodies for 2 h at room temperature. A rabbit anti‐chicken IgY (Texas Red, 1:500, Abcam, Cat# ab6751), goat ant‐rabbit IgG (Alexa Fluor 568, 1:1000, Thermo Fisher, Cat#A11036), and goat ant‐rabbit IgG (Alexa Fluor 488, 1:1000, Abcam, Cat#ab150077) were used as the secondary antibody, and stained with Dapi (Sigma‐Aldrich; Cat# D‐9542) for 10 min.

### 4.6 Senescence‐associated β‐galactosidase staining

SA‐β‐gal is a biomarker of cell senescence. To perform SA‐β‐gal staining, mice brain sections which were immunostained by O4 antibody and photographed and were washed thrice with LacZ washing buffer containing 2 mM MgCl2, 0.01% Na Deoxycholate, and 0.02% Nonidet P‐40 for 5 min. Consequently, the slides were stained overnight with freshly prepared LacZ staining solution at 37°C (Debacq‐Chainiaux et al., 2009). LacZ staining solution contains 40 mM citric acid/Na phosphate buffer pH:5.6, 5 mM [K4Fe (CN)6. 3H2O], 5 mM K3[Fe (CN)6], 150 mM NaCl, 2 mM MgCl2, and 1 mg/mL X‐gal (Bio Basic, Cat#BB0083) (*n* = 3). The images captured from the light microscope were converted to fluorescent images with Image J software adjustments and combined with O4‐stained images.

### 4.7 FluoroMyelin staining

FluoroMyelin™ Red is a selective fluorescent dye used to visualize myelin. OCT‐frozen sections of three mice were washed with PBS and fixed with 4% PFA for 10 min. Slides were stained with FluoroMyelin™ Red (1:300, Thermo Fisher, Cat# F34652) for 30 min at room temperature. Images were quantified blinded using ImageJ software (NIH.USA). The intensity of FluoroMyelin staining in the corpus callosum (CC) area was measured in sections. The staining intensity relative to the intensity of the myelinated area in the Control group was reported.

### 4.8 Luxol fast blue staining

LFB staining was utilized to evaluate the extent of demyelination according to the previous reports (Rayatpour et al., 2022). The fixed sections of three mice were rehydrated and stained with 0.1% LFB (Solvent Blue 38, Sigma, CAS# 1328‐51‐4) at 60°C for 1 h. The extra dye was removed with 95% ethanol, and adequate contrast was achieved with 0.05% lithium carbonate, then washed in distilled water. To visualize the nuclei, 0.1% Crysel Fast Violet (Merck, Cat# K2247947) was used for 3 min. After removing excess dye with distilled water, sections were dehydrated in graded ethanol series, cleared in xylene, and coverslipped with entallan. The extent of demyelination was quantified with ImageJ software. Each section's proportion of the demyelinated area based on the defined threshold level to the whole CC was measured and averaged for each animal and then reported relative to the Control group as the reference.

### 4.9 Electron microscopy

Mice were deeply anesthetized and perfused with precooled 0.1 M sodium cacodylate buffer. The CC with minimum surrounding tissue was immediately collected and immersed in a precooled fixation buffer (2.5% glutaraldehyde, 0.1 M sodium cacodylate buffer) for 2 h at 4°C. Fixed tissue was washed twice in 0.1 M sodium cacodylate buffer, followed by postfixation in 1% osmium tetroxide for 90 min. Next, the samples were serially dehydrated in ethanol and embedded in Agar resin (Agar Scientific Ltd) to prepare the blocks. The blocks were then sectioned into 50 nm ultrathin sections using an ultramicrotome type 4801A (LKB‐producer AB‐Stokholm) and placed on 300 mesh copper grid and double stained with 20% lead citrate and uranyl acetate for electron microscopic imaging using a transmission electron microscope (Hitachi HT‐7800).

### 4.10 Toluidine blue staining

Toluidine blue staining was performed as mentioned previously (Seyedsadr et al., 2019), with some modifications. Resin‐embedded blocks were sectioned 1–2 μm thin by an ultramicrotome (C. Reichert, Austria). Sections were stained with 1% toluidine blue, and the excess dye was rinsed with deionized water; slides were dried at room temperature and evaluated under Olympus light microscopy BX‐51. Images were used to calculate the g‐ratio (the ratio of the bare axon diameter to the myelinated axon). For each mouse, three separate sections were used for analysis. Each group included three mice. The frequency of remyelinated axons and the g‐ratios of these axons were compared.

### 4.11 RNA extraction and RT‐qPCR

Mice were euthanized, and following brain harvesting, the brains were immediately sliced every 2 mm using a brain matrix. The corpora callosa were then dissected from the brain slices. Subsequently, the tissue fragments were snap‐frozen in liquid nitrogen and preserved at −80°C until use. Every two mice's CC fragments were pooled together for total RNA extraction using the RNA isolation kit based on the manufacture protocol (RiboEx solution, Gene All, Cat# 301–001). The RNA quality and concentration were assessed using a nano spectrophotometer (Thermo Scientific). Samples were stored at −80°C till cDNA was synthesized. About 200 ng of total RNA was reverse‐synthesized to cDNA using a reverse transcription Kit (Pars Tous Biotechnology, Cat# A101161). Next, the cDNA was used to quantify the RNA expression ratio of the desired genes using RealQ plus 2× master mix green (Ampliqon, Cat# A325402) on a Rotor‐Gene device (Qiagen, Germany). The primers used in this study are presented in Table 1 (Sina Colon). All reactions were performed in duplicated and analyzed using the 2^−ΔΔCT^ method. GAPDH gene expression was utilized as a reference gene for quantification.

**TABLE 1:** The sequence of the primers used in this study.

Gene name	5′‐forward primer‐3′	5′‐reverse primer‐3′
*GAPDH*	CATCACTGCCACCCAGAAGACTG	ATGCCAGTGAGCTTCCCGTTCAG
*Icam1*	GAGGTGGCGGGAAAGTTC	CAGTCCGTCTCGTCCAGC
*Il6*	TCCGCAAGAGACTTCCAGC	TGTGAAGTCTCCTCTCCGGAC
*Cdk‐2*	GCTCGACACTGAGACTGAAGG	GACATCCAGCAGCTTGACG
*Pcna*	CGAAGCACCAAATCAAGAGAA	*CAGCTGTACTCCTGTTCTGGG*
*P16*	TGGACCAGGTGATGATGATG	TCGAATCTGCACCGTAGTTG
*P21*	CTGAGCGGCCTGAAGATTC	AGAAATCTGTCAGGCTGGTCTG
*Trp53*	CCGCCGACCTATCCTTACC	TCTTCTGTACGGCGGTCTCTC
*Tert*	GCAGCCTGTTTGACTTCTTCC	GCGTATAGCACCTGTCACCAA
*Tgfbr2*	GCTGTGGGAGAAGTGAAGGA	ATGCTCTCCACACAGGGGT
*mbp*	CCCTCAGAGTCCGAC	GCACCCCTGTCACCG
*plp*	TACGGCAATTAGGGAGCACA	TCTCAGCTCCTTGGAAACCA

### 4.12 Genomic and mitochondrial DNA extraction and analysis

Comparing the ratio of mitochondrial to nuclear DNA (nDNA) is a method used to evaluate the integrity of nuclear and mitochondrial genomes exposed to various genotoxins, particularly for detecting damages induced by reactive oxygen species in mitochondrial DNA (mtDNA) (Dolcini et al., 2020). For this assay, three mice brain tissues were homogenized separately, and DNA was isolated using the standard phenol/chloroform extraction method. DNA quantitation was performed using a nanodrop spectrophotometer (Thermo Scientific). Samples having 260/280 absorbance ratios ≥1.8 were considered pure DNA. The DNA integrity was verified through agarose electrophoresis. RT‐qPCR was employed for both nDNA and mtDNA quantification using RealQ plus 2× master mix green (Ampliqon, Cat# A325402), on a Rotor‐Gene device (Qiagen). The primer sequences for mtDNA were 181 bp mitochondria fragment (5′‐ CAAACCTTTCCTGCACCTCC‐3′) and mtR3319 (5′‐ AGGCGTTCTGATGATGGGAA‐3′) and those for nDNA, used for loading normalization were 3′,5′ cyclic AMP phosphodiesterase F (5′‐GTTCCCGCCTTCTTCCTCTG‐3′) and 13′,5′ cyclic AMP phosphodiesterase R (5′‐ GTTTGCTTGCCGACTCCTTG′) (Gonzalez‐Hunt et al., 2016). The PCR profile consisted of an initial 2 min heating at 50°C, followed by 10 min heating at 95°C, and then 40 cycles of amplifications (15 s 95°C, 60 s 60°C). The mtDNA/nDNA ratio can be calculated by 2^ΔΔCT^.

### 4.13 Biochemical assays

Brain tissues were homogenized in 50 mM potassium phosphate buffer (*p*H = 7) containing 1 mM Ethylenediaminetetraacetic acid (EDTA) and 100 mM phenylmethyl sulfonyl fluoride (PMSF) (Sigma‐Aldrich, Cas# 329–98.6). The homogenate was then centrifuged at 12000 *g* (Hitachi) for 15 min at 4°C. The resultant supernatant was utilized for biochemical analyses. The protein concentration of each homogenate was determined using the Bradford method and used for normalizing biochemical readouts.

Malondialdehyde (MDA) is an indicator of free radical production and oxidative stress. MDA was quantified spectrophotometrically in tissues with an assay based on Thiobarbituric acid (Draper & Hadley, 1990). The homogenate samples were mixed with 0.5 mg/mL butylated hydroxytoluene and 10% trichloroacetic acid for 1 h at 100°C, then centrifuged at 1600 *g* for 10 min at room temperature. The supernatants were mixed with 0.07% w/v Thiobarbituric acid at 95°C for 1 h, again at 100°C. Subsequently, the absorbance was measured at 532 nm by an ELISA plate reader (Bio Tek). (*n* = 5–6).

The oxidative stress index (OSI) was defined as the ratio of the total oxidant status (TOS) to the total antioxidant capacity (TAC) level. Specifically, OSI is defined as TOS (μmol H_2_O_2_ Eq/L)/TAS (μmol uric acid Eq/L). TOS was measured according to the Erel TOS method (Erel, 2005). This method is based on converting ferrous ions to ferric ions in the presence of oxidative species. Briefly, 25 μL of the homogenate samples were mixed with 225 μL of Ferrous Oxidation‐Xylenol orange (FOX) reagent. After 30 min incubation at room temperature, their absorbance was measured at 580 nm using the ELISA plate reader. The FOX reagent contains 100 μL of 25 mM FeSO_4_ in 2.5 M H_2_SO_4_, 500 μL of 125 mM xylenol orange in methanol, 9 mL of 1 M sorbitol in ultra‐pure water, and 400 μL ultrapure water. TAC level was quantified based on the Erel TAC method (Erel, 2004). In brief, 5 μL of the homogenate samples were mixed with 195 μL of ABTS^•+^ reagent containing 7 mM ABTS and 2.45 mM potassium persulfate. After 6 min incubating at 30°C, the decline in the absorbance was measured at 734 nm using the ELISA plate reader (*n* = 5–6).

Reduced glutathione (GSH) was measured following an established procedure (Jollow et al., 1974). To remove proteins from the samples, 0.5 mL brain homogenate was precipitated with 1 mL of sulphosalicylic acid (4% w/v). The precipitate was removed by 10 min centrifugation at 10000 *g*. Then, 1 mL of the sample supernatant was combined with 0.1 Ellman's reagent (5,5′‐dithiobis‐(2‐nitrobenzoic acid) (4 mg/mL) and 0.9 mL tris–HCl (0.1 M, *p*H 8). The absorption value was measured at 412 nm using ELISA plate reader (*n* = 5–6).

### 4.14 Statistical analysis

Data analyses were performed in a blinded manner. Statistical analysis was performed using GraphPad Prism v.9.0.0 (GraphPad Software, San Diego, CA, USA) software. One‐way analysis of variance (ANOVA) with Tukey's multiple comparison test was used to compare multiple data points. Two‐way ANOVA with Tukey's multiple comparisons was used for the analysis of mitochondria count. Data from myelin thickness axon diameter, and mitochondrial length were analyzed by the Kruskal–Wallis test followed by Tukey's multiple comparison test. *p* values <0.05 were considered statistically significant. All values are given as mean ± SEM (standard error of the mean).

## AUTHOR CONTRIBUTIONS

E.P, SH.A, and M.J. planned and designed the study. E.P performed and analyzed experiments and drafted the manuscript. SH.A and M.J supervised the experimental program. M.J. and SH.A finalized the manuscript. E.P and M.S performed ultrastructural study and analysis. All authors confirmed the final manuscript.

## FUNDING INFORMATION

This study was supported by Research Council of the University of Tehran (grant number. 6401007/6/34), the University of Tarbiat Modares (grant number. NIMAD 971403), and the Iran National Science Foundation (INSF) (grant number. 400234).

## CONFLICT OF INTEREST STATEMENT

Authors declare no conflict of interest.

## Data Availability

The data that support the findings of this study are available from the corresponding author upon reasonable request.
